# A Study of Staff Experiences of Delivering Prehospital Emergency Services in India in the Context of the EMS Agenda 2050 Objectives

**DOI:** 10.1155/emmi/4265123

**Published:** 2026-10-30

**Authors:** Vipul Vinzuda, Niall Deloughry, Linda Shore, Leonard O’ Sullivan

**Affiliations:** ^1^ School of Architecture and Product Design University of Limerick Limerick Ireland ul.ie; ^2^ Transportation and Automobile Design National Institute of Design Gandhinagar India; ^3^ Interdisciplinary Research Emerging Technologies The Glasgow School of Art Glasgow UK; ^4^ School of Architecture and Product Design Health Research Institute Limerick Digital Cancer Research Centre University of Limerick Limerick Ireland ul.ie

**Keywords:** emergency medicine, health technology, low- and middle-income country, prehospital care, thematic analysis

## Abstract

**Background and Objective:**

Emergency care services are crucial to the delivery of modern healthcare. This study investigated prehospital staff experiences in delivering emergency care in a low‐ to middle‐income country (LMIC) India. The study was guided by the EMS Agenda 2050 principles for people‐centred and technology‐enabled health systems. The study was performed with emergency medical staff from the city of Gandhinagar, India, which is designated as a future smart city.

**Method:**

This research used purposive and snowball sampling with 23 participants (ambulance drivers, emergency medical technicians, paramedics and first‐aiders) from government, non‐governmental and private organisations. Online semistructured interviews explored current prehospital care practices, challenges and opportunities. NVivo software was used for qualitative analysis, with a reflexive thematic analysis approach used to derive themes and present findings.

**Results:**

The thematic analysis identified four primary themes with subthemes: (1) job satisfaction and challenges, (2) attitudes and emotions, (3) technological factors and (4) efficiency and caring. Findings highlight opportunities for technological interventions to enhance citizen awareness and training, support relatives during emergencies, provide bystanders with medically safe guidance and facilitate digitalisation in prehospital care.

**Conclusions:**

This research advances understanding of the human and operational realities of prehospital care in a future smart‐city context. Healthcare professionals demonstrated expertise and empathy in managing complex emergencies while recognising the importance of their role in caring for critically ill and injured patients. Although digital technologies are increasingly integrated into practice, hands‐on skills and experiential judgement remain fundamental to effective care. The findings underscore the need for stakeholder collaboration, enhanced professional training and innovative citizen awareness initiatives to strengthen resilient prehospital care systems.

## 1. Introduction

There is a pressing need to strengthen prehospital care, a crucial component of emergency medical services (EMS), that provides initial treatment and transport for patients during emergencies [1]. The evolution of healthcare systems requires innovative approaches to enhance EMS and meet future emergency care demands [2]. The World Health Organisation (WHO) emergency care system framework defines essential functions of emergency response systems, including governance, infrastructure, communication and legal mandates. The framework enables countries to assess their system capacity and to design context‐specific plans to strengthen time‐sensitive services [3]. Transformation and innovation are central to continued improvements in EMS to meet the needs of growing populations and to increase the quality of service provided. Developing patient‐centred prehospital EMS, particularly through technological advancements in rapidly urbanising areas, is increasingly important [4].

EMS in low‐ and middle‐income countries (LMICs) face systemic challenges [5]. Critical gaps in infrastructure, training, financial limitations and governance can adversely affect service delivery, impacting response times and consistency of care [6]. Fragmented EMS further weaken coordination and service continuity [5]. The WHO reports that approximately 134 million adverse events occur annually in LMIC healthcare settings, contributing to an estimated 2.6 million deaths [7]. The burden of unsafe care resulting from limited infection control, hierarchical culture and deficient legal frameworks inadvertently affects patient safety [6]. There is an urgent need for investment in EMS infrastructure, training, governance and community engagement to deliver high‐quality prehospital care [8].

By 2035, India, as an LMIC, is expected to experience further rapid urbanisation with more than 675 million people residing in cities [9]. This growth will create pressure on healthcare services that have previously received criticism for inconsistent quality care, a lack of trust and inadequate public awareness [10]. Indian traffic congestion affects ambulance movement, which remains a challenge for prehospital care services in cities. India has advanced plans to develop acute and emergency services further [11]. By 2035, the vision is to create a predictive, responsive, integrated, tiered public health surveillance system, leveraging technology, data analytics and a citizen‐centric approach with rapid response teams to achieve optimal patient outcomes [12]. An optimal outcome means safe, effective emergency care, whereas a resilient city adapts and recovers with human‐centred, tech‐enabled systems. Many cities in India are experiencing very high rates of urbanisation due to population growth and migration. India has designated 100 smart cities of the future, which include digital health initiatives under the Smart Cities Mission with the aim of enhancing urban governance and public services through technology‐driven solutions and integrated digital infrastructure. The National Digital Health Mission (also known as Ayushman Bharat Digital Mission) seek to establish a unified digital health ecosystem through interoperable platforms and electronic health records, enabling accessible, patient‐centred care [13].

The EMS Agenda 2050 is a visionary framework that outlines six guiding principles to shape the transition of future EMS systems from traditional transport‐focused models to holistic proactive care systems. These principles are (1) inherently safe and effective, (2) integrated and seamless, (3) reliable and prepared, (4) socially equitable, (5) sustainable and efficient and (6) adaptive and innovative [14]. The EMS Agenda 2050 aims to promote technology integration, data‐driven operations and inclusive governance to balance patient outcomes and the well‐being of healthcare staff. Although the EMS Agenda 2050 was developed for high‐income contexts, its principles are highly relevant for LMICs with aspirations for technology growth and innovation, such as in smart cities [15–17].

The objective of this research was to conduct a qualitative study of EMS staff’s experiences and opinions regarding challenges in delivering prehospital care in the city of Gandhinagar, a candidate smart city of the future in India. The study is informed by the EMS Agenda 2050, a visionary framework that emphasises people‐centred care, safety, adaptability and technology‐enabled decision‐making in the development of resilient prehospital care systems.

## 2. Method

### 2.1. Study Design and Participants

A qualitative exploratory study was conducted using semistructured interviews grounded using interpretive ontology to recognise the varied experiences of participants involved in prehospital care. This study adopted an exploratory approach to examine EMS professionals’ perspectives on current challenges, future opportunities and emerging technological developments relevant to prehospital care in Indian smart cities.

In the Indian EMS context, emergency medical technicians (EMTs) are typically trained to provide basic life support (BLS), whereas paramedics are trained to deliver more advanced prehospital interventions, including advanced life support (ALS). Paramedics, EMTs or ambulance drivers typically attend to medical emergencies and thereafter transport patients to hospital emergency departments for stabilisation and treatment. The focus of this current research was on prehospital healthcare professionals. Perspectives of in‐hospital staff are addressed in a separate study by the current authors.

Purposive sampling was initially used, followed by snowball sampling to connect with and recruit the study participants. EMS staff from government hospitals, multispeciality hospitals, private clinics and private ambulance operators were invited to participate in this study. Additionally, staff members responsible for operating ambulance services under the public–private partnership (PPP) model with the state government also participated in the study. Contacts were established from the official websites and publicly available details of respective organisations, institutions and individuals across the city. A total of 29 individuals were invited to participate in the study, of which 23 indicated their availability and interest. All participants met the inclusion criteria of being emergency medical professionals primarily working in the city. Interviews were conducted in their preferred language, either Hindi or Gujarati. All participants provided informed consent to participate in the research study by signing the consent form electronically.

The Research Ethics Committee of the University of Limerick approved this study (Reference No. 2021/06/03). Table 1 summarises the participants’ demographics and background. There were 5 female and 18 male participants. Roles included ambulance drivers, EMTs, paramedics and first‐aiders, with experience ranging 5–15 years. Participants worked in private/multispeciality government hospitals in the emergency department or ambulatory service.

**TABLE 1:** Summary of participants’ demographics and backgrounds (*N* = 23).

Details		*N*
Age range (years)	21–29	8
	30–39	5
	40–49	7
	50–59	2
	60–69	1

Gender (F:M)	5:18	

Organisations	Multispeciality hospitals	3
	Private ambulance owners	7
	Government‐funded ambulances (108)	12
	A voluntary organisation	1

Professional role	Ambulance driver	10
	EMTs (basic life support trained)	10
	Paramedic (advance life support trained)	2
	First‐aider	1

### 2.2. Data Collection—Semistructured Interviews

The study utilised semistructured interviews to obtain information on prehospital care professionals’ experiences, as per the study objectives. The interview structure and questions were based on the six guiding principles of the EMS Agenda 2050 [14]. These principles informed eleven open‐ended questions (Table 2).

**TABLE 2:** Set of semistructured interview questions.

Questions
1. When enroute to a critical emergency situation, what type of information do you receive and how do you/immediate team cognitively and emotionally prepare to provide the best treatment to meet the clinical care needs of the patient(s)?
2. What are the social barriers (for example, cultural, economic, educational, social) that affect patients receiving the highest standard of clinical care to vulnerable patients at a critical emergency?
3. What are the difficulties/challenges you experience while engaging with members of family/public/bystanders at a critical emergency?
4. When treating an emergency patient at a critical incident, what do you personally regard to be effective care?
5. What are the elements of a prehospital care EMS system that can be improved to deliver appropriate, transparent, and efficient critical emergency care?
6. What are the difficulties/challenges you experience while engaging with other professionals at a critical emergency?
7. What are the challenges in adhering to all safety precautions while delivering care to patient(s) in a critical emergency?
8. What challenges/difficulties do you experience in delivering highest standard of care every time?
9. How could the current EMS system be made adaptable to dynamic critical emergency scenarios?
10. What digital technologies (for example, telemedicine) do you currently use to assist in providing prehospital care?
11. How could technologies and innovation be used to transform the delivery of prehospital emergency healthcare for citizens in smart cities of the future?

Interviews were conducted online using a videoconferencing platform. The entire study was conducted online due to the global COVID‐19 pandemic during the years 2021–2022. In advance of the interviews, each participant received an email link to complete an online consent form. All interviews were completed with only the interviewer and participant present. All interviews were conducted by a single researcher (VV) to ensure consistency throughout the study. The interviewer adhered to the questions as a guide, asking follow‐up questions as conversation flowed to gain a deeper understanding of the responses. Interviews were approximately 60 min in duration (minimum 11.13 and maximum 73.04 min). Consistent with the thematic analysis (TA) approach, data collection continued until the dataset was considered sufficiently rich and diverse to support meaningful theme development. Subsequent interviews primarily added depth and contextual understanding to existing themes, providing confidence that the study objectives had been adequately addressed and that the dataset captured a broad range of EMS perspectives and experiences.

Over 1020 min of interviews were recorded and transcribed verbatim. Three interviews were conducted in Hindi and 20 in Gujarati. These interviews were translated into English by the trilingual first author and reviewed against the original transcripts during analysis to ensure accuracy, retention of intended meaning, contextual and cultural nuances and conceptual equivalence. Observations regarding body language, facial expressions, tone of voice and other notable aspects were recorded in the interview notes. Nonverbal signals provide valuable context, enabling deeper comprehension and aiding the development of a more nuanced, interpretative analysis of the findings. Each audio file and transcript was examined to ensure accuracy and consistency. To maintain confidentiality, all participants were anonymised and assigned a pseudocode to identify each data file. Formal member checking was not undertaken; however, trustworthiness was enhanced through reflexive memoing, iterative engagement with the data and regular discussions among the research team throughout coding, theme development and interpretation. Reflexivity was maintained through ongoing critical reflection and regular discussion with the research team throughout data collection, analysis and interpretation.

### 2.3. Data Analysis

The research followed a qualitative research methodology, which provided an opportunity to develop rich insights captured from the depth and context of participants’ experiences, perspectives and expressed feelings. TA was used as an inductive qualitative data analysis approach, owing to its strengths of reflexive flexibility and ability to explore rich datasets with depth [18].

To demonstrate rigour, the TA process aligned well and allowed time for several rounds of review and reflection to interpret the data. Additional methods of writing remarks, making notes on transcripts, tabulating data in Excel, using affinity diagrams, mind maps and flowcharts were used to further assist in interpreting the data. This provided a systematic approach for organising and analysing interview data, including identifying themes (Figure 1).

**FIGURE 1:**
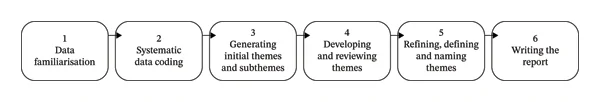
Thematic analysis process: six‐phase approach (Braun V et al., 2019).

All data were uploaded to NVivo 14 (Lumivero—formerly QSR International) for coding, and additional data and audio files were anonymised and securely stored on a server. All transcribed interviews were imported to NVivo for line‐by‐line coding. In total, 1148 codes were generated from the 23 semistructured interviews.

The analysis followed a six‐phase comprehensive approach, starting with data familiarisation, during which the dataset was repeatedly read to gain a thorough understanding, and key observations and important statements were noted. Next, systematic data coding was carried out collaboratively by researchers VV and LS, with initial codes highlighted and organised using Microsoft Excel sheets, with short codes and descriptive texts. NVivo software was then employed to generate initial themes by locating, classifying and organising the data, codes and quotes. This process involved searching for themes and subthemes to identify repeating patterns through code comparison and review the development and review of themes involved arranging data with similar interpretations into themes and subthemes. Miro software was used to create affinity diagrams to identify relations and connections within the data. Refining and defining the themes resulted in a final set of four themes derived from 10 subthemes, each clearly articulated to convey its respective story yet distinctly unique. In the final stage, these themes were linked to the literature, research questions, coding extracts and final theme definitions. While themes were generated inductively through reflexive TA, they were subsequently interpreted in relation to the six EMS Agenda 2050 principles to explore their relevance to the Indian EMS context. This enabled the identification of areas of alignment, contextual gaps and opportunities for future EMS development in India.

## 3. Results

Four themes and 10 subthemes emerged from the analysis (Figure 2 and Table 3). The results from coding were organised into four broad themes, each with subthemes and supporting quotes.

**FIGURE 2:**
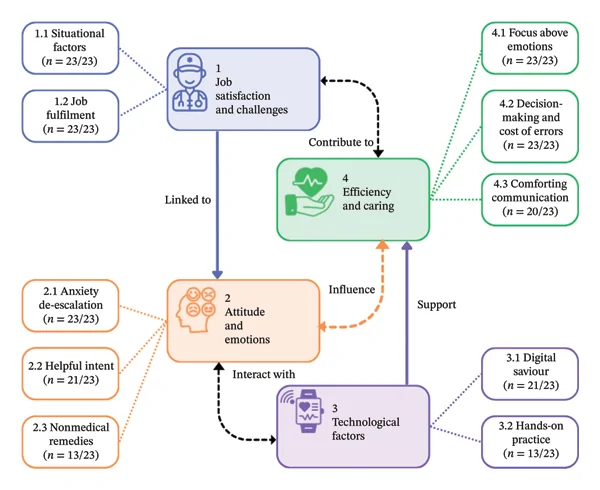
Thematic map illustrating participant counts and reciprocal relationships among the four themes. (1) Job satisfaction and challenges are linked to (2) attitudes and emotions, whose interactions with (3) technological factors influence and support (4) efficiency and caring prehospital care. Experience of care contributes to job satisfaction and shapes responses to workplace challenges, creating a continuous and interconnected cycle within prehospital practice (solid arrows indicate directional influence or contribution between themes, while bidirectional arrows represent reciprocal and mutually reinforcing relationships).

**TABLE 3:** Summary of the four main themes.

Themes	Definition
1. Job satisfaction and challenges (*n* = 23)	The theme captures the dual sides of out‐of‐hospital emergency service, where professionals face daily unpredictable behaviours, social barriers, and logistical issues. Despite these obstacles, the pride and contentment they derive underscore their motivation, satisfaction, and sense of purpose in saving critical patients as a duty. ^∗^ *Interpretive commentary: Making a meaningful difference can sustain job satisfaction, resilience, and self-motivation amid operational challenges.*

2. Attitudes and emotions (*n* = 23)	Bringing a shift in people’s attitudes to safeguard an emergency patient, responding more sensibly, managing anxiety, acquiring new skills, and learning know‐how to be a helpful citizen. ^∗^ *Interpretive commentary: Enhanced public awareness and preparedness can promote more confident, responsible, and supportive citizen responses during emergencies.*

3. Technological factors (*n* = 23)	The integration of digital technologies, including smartphones, GPS navigation, video calling, and mobile applications, enhanced communication, data sharing, and operational efficiency, while highlighting the continued importance of training, professional skills, and human judgement alongside technology. *∗Interpretive commentary: Digital innovation can enhance efficiency, while human expertise and adaptive judgement remain central to effective emergency care.*

4. Efficiency and caring (*n* = 23)	Efficient care involves empathetically addressing critical patients’ medical needs with optimism and pragmatism while staying alert to any human error. It ensures seamless workflows and proficient resource management for ideal patient outcomes. ^∗^ *Interpretive commentary: Integrating empathy, clinical attentiveness, and operational efficiency appears central to achieving positive patient outcomes.*

^∗^Interpretive commentary developed by the researchers to highlight the underlying meaning of participants’ accounts.

### 3.1. Theme 1: Job Satisfaction and Challenges

This theme highlights how experiences of helping patients in critical situations contributed to job satisfaction, resilience and a strong sense of purpose despite the uncertainties and challenges of prehospital care, reinforcing the importance of meaningful work in sustaining EMS professionals.

#### 3.1.1. Situational Factors

Situational factors in prehospital care require professionals to adapt to unpredictable scenarios and rapidly changing patient conditions while balancing competing demands and delivering timely en route care. This underscores the importance of adaptive expertise in navigating complexity and uncertainty.

‘Sometimes public gives way, but otherwise the drive is very stressful. There is a lot of pressure, and we keep thinking about “how quickly we can reach the patient?” By the time we arrive at the scene and take the emergency patient, our breathing starts to settle down’.—P2

Patients’ and bystanders’ behaviours during emergencies can create additional stress and hinder care delivery, as EMS professionals must manage difficult reactions, emotional distress and unsafe behaviours while providing timely care and transport.

In the monsoon season, it’s a challenge. When transporting a patient, there are a few problems, especially in a new area where we have no idea about the route.—P7

The participant highlighted situational challenges, including infrastructural limitations such as unreliable communication networks, public attitudes, low or no awareness, contextual factors such as festivals, weather conditions, geographical constraints, high‐rise buildings, time pressures and traffic congestion, all of which hindered timely prehospital care delivery.

‘We brought a patient’s relatives to the hospital and reunited them. Reconnecting an unidentified patient with their family is a difficult job, sir. Finding contact numbers, contacting people, dealing with police cases, we have to go through all of these things’.—P18

‘If the patient does not have a relative with them, private hospital do not take such risks. They may say, “he has no one with him; take him to a government hospital instead”’.—P19

Managing unidentified patients without relatives required additional effort, while patient and family preferences often complicated care decisions. Participants highlighted that uncertainty, hospital refusal of unidentified patients, socioeconomic constraints, limited awareness and belief‐based barriers created challenges beyond clinical control, potentially delaying or compromising prehospital care.

#### 3.1.2. Job Fulfilment

Positive patient impact and social recognition reinforced self‐motivation, professional fulfilment and sustained commitment to prehospital care, contributing to workforce resilience in demanding emergency settings.

‘Since joining 108, we have been given the title of “pilot”. Just like, a pilot flies an aircraft in the sky, we are pilot on the ground instead, and we have been given that honor’.—P18

They derived a sense of contentment from performing their roles sincerely, gained social recognition and a distinct professional identity and viewed serving the community as second nature.

‘Even if my organisation told me, “Brother, you will not be paid,” I would still want to come to work. Sir, I do not want to take leave; I want to work’.—P12

Positive aspects centred on the appreciation and recognition received for providing emergency medical care at the scene, along with a strong sense of purpose and work satisfaction.

### 3.2. Theme 2: Attitudes and Emotions

The mismatch between citizens’ willingness to help and their preparedness to respond effectively highlighted public behaviour and awareness as critical contributors to safe and effective prehospital care.

#### 3.2.1. Anxiety De‐escalation

Anxiety and uncertainty among relatives and bystanders often complicated emergency decision‐making, positioning EMS professionals as both clinical responders and coordinators of emotionally charged situations.

‘Sometimes it is not the same case. For example, if there has been an accident and we have not yet arrived, the public often creates panic, saying, “He is going to die; he will not survive.” Even if the poor fellow is not dying, such panic can make the situation worse’.—P13

Calm demeanor, confidence, medical reasoning and composure in the presence of large crowds were identified as essential skills. These qualities, together with situational awareness and professionals’ focus, helped reduced public anxiety and support effective emergency care delivery.

‘We should give an impression of confidence from head to toe, showing that we will give more than 100%. Such assurance should be noticeable in our appearance and in the way we conduct ourselves’.—P16

‘The way you speak, your goodwill, and even a glance reflect your perspective. It is not our way to say in a harsh tone, sir, things like, “Can’t you see? Don’t you understand?”’.—P20

‘Whatever is destined to happen will happen. As a Rajput (warrior community), I have always had the passion and resolve to help any patient under any circumstances. If we keep our minds under control, nothing will happen to us’.—P1

#### 3.2.2. Helpful Intent

Humanitarian values extend beyond professional obligations, fostering a commitment to equitable care regardless of a patient’s identity or circumstances.

‘Whatever a person’s caste or social status, whether they are working class, rich or poor, or a prominent contractor, when they are on the road, our first priority is to take them to a civil hospital’.—P1

Professionals relied on their training, experience and support from doctors to deliver optimal care at the scene and during transport. However, bystanders’ behaviours such as inaction, overenthusiasm, urging staff to rush, unsafe patient handling or leaving the scene after making an emergency call often hindered an effective response.

‘While we are enroute, they are gone. As a result, the service cannot be provided. When a patient needs assistance but there is no confirmed address or clear location, reaching them becomes difficult. People have left the scene. Due to this, patient can’t receive treatment soon’.—P19

The findings indicated that limited public awareness contributed to uncoordinated bystander involvement, highlighting the need for citizen training to channel helpful efforts into practical support.

#### 3.2.3. Nonmedical Remedies

Limited public understanding and emergency preparedness emerged as important yet under‐recognised factors in effective prehospital care, requiring EMS professionals to navigate the intersection of medical advice, cultural beliefs and community behaviours.

‘We have to transport patients from one place to another, but we also have to explain things to them. We say, “Brother, don’t do this. Keep both medicine and *dua* (prayer) together. First, get the treatment, and then go there.” Some people understand, and some do not, That’s how it is’.—P17

‘We tell people not to give anything by mouth or make the patient smell onions or anything similar. This is part of educating the public through our pre‐arrival instructions, as some people in Gandhinagar have such understanding. We tell them, “Brother, don’t do such things on your own until we reach the scene”’.—P19

### 3.3. Theme 3: Technological Factors

This theme highlights the complementary role of digital technologies, such as smartphone applications, which enhance communication and coordination in supporting emergency care, while reinforcing the enduring value of hands‐on expertise and established life‐saving practices.

#### 3.3.1. Digital Saviour

Most participants had readily integrated digital technologies into their routine workflows, using them for sharing patient vitals, teleconsultations, location tracking, paperless documentation and timely recording of emergency scenes.

‘We often take photographs or make a record of anything serious that happens inside the ambulance. For example, there are unruly patients whose behaviour needs to be documented’.—P22

Smartphone applications were also used for training and revision, while ambulance service providers shared professional practice updates with staff.

‘Every year, we learn new things, and new changes keep happening. We continue to adapt, and a lot has changed for the better’.—P13

#### 3.3.2. Hands‐on Practices

The importance of hands‐on skills, resourcefulness and experience‐based decision‐making remained central to emergency care despite technological advances and practical challenges such as technical disruptions, limited awareness, high costs and variable service quality.

‘There are problems with coverage, and another thing, sir, is that what I can explain through audio, I cannot always explain through video. If you want to see, sir, you can see what I am doing’.—P20

Inconsistent internet coverage and technical errors can hinder care, and some staff found video calls distracting. Many rely more on experience and tacit knowledge, trusting their skills, training and protocols over tools such as GPS or defibrillators during crises.

‘Technology is helpful, but when there is an emergency, you do not remember the technology. Your hands simply take over’.—P8

The findings reveal that technological innovation is most valued when grounded in practical experience and used to strengthen, rather than replace, human expertise and patient‐centred care.

### 3.4. Theme 4: Efficiency and Caring

Life‐saving prehospital care relies as much on empathetic and adaptive decision‐making as on clinical skills, safety and operational coordination.

#### 3.4.1. Focus Above Emotions

Timely and effective prehospital care depends on balancing compassionate care with focused, objective decision‐making and coordinated action under pressure.

‘I have only one motivation: to be as fast as possible for every patient. Whether they are poor or rich, going to a private or a government hospital, my only priority is to be the fastest, at every time and under any circumstances’.—P1

‘I believe in seconds, not minutes or hours. Saving lives depends on seconds. Emergency patients often have only a few seconds, not minutes, and we have to make use of those seconds’.—P12

Prioritising patient survival from controlling bleeding to clearing airways require safety to be treated as a top priority, with staff emphasising scene, personal and vehicle safety as a routine practice before providing care or seeking additional support.

‘We ensure our own safety first before going to the scene, sir. If we can see from a distance that the scene is unsafe, we do not proceed, because if our ambulance is not safe or we ourselves are not safe, we will not be able to help the patient’.—P18

Professionals prioritised patient treatment over the emotions or behaviours of families, bystanders or patients, maintaining a life‐saving focus. Long service experience helped build the mental preparedness and foresight required for such a role.

‘When we arrive, some might be angry at us, but we never think about that. Our focus is only on the patient, what the emergency is and how to manage the patient’.—P23

‘As the days and years went by, we encountered many patients, their families, and many types of emergencies. Similar experiences kept occurring, which gave us foresight, as if a third eye had opened’.—P18

#### 3.4.2. Decision‐Making and Cost of Errors

Positive patient outcomes depend not only on clinical expertise, but also on proactive risk management, public awareness, system preparedness and continuous service improvement, as avoidable errors could contribute to patient distress, delays in care and wider adverse consequences.

‘Often it happens; people become emotional, and often safety is overlooked. […] In a road accident, many bystanders come forward, and if they are not properly trained, the patient can suffer because of it. I have experienced this myself’.—P10

Participants experienced difficulties when people overlooked safety in emotionally charged situations, leading to infections and hindering further patient care. Human errors during critical stages of prehospital emergency care often stemmed from overconfidence, interpersonal tensions, financial stress, night shifts, fatigue and varying levels of training.

‘Many times, staff become overconfident. That is why I ask, so that it does not happen to us. “If you don’t know, don’t give wrong advice”’.—P6

Participants revealed scope to enhance government‐operated emergency services, noting that ambulance hygiene, equipment, space and location critically affect care. They stressed the need to strengthen staff resilience, consolidate resources and modernise EMS to ensure safe, well‐equipped transport supported by trained professionals.

‘Someone may simply drive the patient in their own car, that is parked in the house, or a nearby car, and even reach the hospital before the 108 ambulance. However, they may not know which stretcher to use, which ward patient must be taken, or which doctor to consult, and so on’.—P12

‘We cannot risk three lives to save one patient, sir. We transport the patient in a calm and controlled manner, according to the severity of the emergency’.—P18

#### 3.4.3. Comforting Communication

Stabilising patients required more than protocol‐based treatment alone; it depended on EMS professionals’ ability to adapt to changing circumstances, provide reassurance and comfort and respond effectively under pressure both at the scene and during transport, underscoring the importance of adaptive, person‐centred care.

‘If there is a sudden emergency in which a person is injured and suffered, the risk of shock increases. At that time, they need psychological first aid alongside physical first aid’.—P10

‘They are very frightened, we tell them, “Nothing has happened, sir, so don’t worry. You will recover very quickly, and with a little treatment, you will be able to go home as well”’.—P13

Participants highlighted that effective prehospital care extends beyond clinical intervention, with emotional reassurance playing a critical role in relieving doubts and fears, instilling confidence, fostering trust and supporting patients during stressful emergencies.

‘By simply providing reassurance, 50% of a patient’s condition improves. Reassurance is something that motivates every patient’.—P18

Collectively, these findings indicate that transformative and resilient prehospital care is shaped not by technological innovation alone, but by informed citizens, compassionate professionals and adaptive expertise in navigating complex emergencies.

## 4. Discussion

### 4.1. Thematic Findings and Relevance to Service Delivery in an LMIC City

The findings reveal the experiences of EMS staff in delivering care in a large city in an LMIC, offering insights into barriers and opportunities for the development of the Indian emergency care service system, embarking on large‐scale technology adoption through smart cities.

Although technology featured as an important theme, participants consistently situated technological opportunities within broader organisational, social and system‐level contexts, indicating that future EMS development will rely on coordinated advancements across multiple domains.

The resulting themes indicate that professionals at the scene and en route offer empathetic approaches and positivity, despite circumstantial difficulties in low‐income environments. Low levels of awareness and a lack of trained citizens can hinder safe assistance to vulnerable patients at emergency scenes [4]. The study reaffirms the importance of first aid and emergency knowledge among citizens.

Reluctance to seek timely medical help stems from cultural factors associated with fear of financial implications, misconceptions that lead to delays and a preference for traditional remedies [1]. During health crises, individuals often experience a deepening of their faith in a supreme being. While EMS staff respect personal beliefs, they prioritise medical and scientific methods.

Professionals still prefer hands‐on methods and remain cautious while adopting new technologies, due to their individual nontechnical backgrounds or education influencing mindsets. For instance, one private ambulance operator bans smartphones due to potential driver distractions and increased cognitive load. Perceived reliability issues can lead to resistance to adaptation [1]. Current infrastructural limitations and unreliable communication networks can hinder timely prehospital care delivery [4].

The digital divide extends beyond access to technology, encompassing disparities in age, gender, digital literacy, workforce competency and organisational readiness, potentially limiting the equitable adoption, scalability and benefits of digital innovation across EMS systems [19, 20].

### 4.2. Future Vision for EMS Prehospital Systems

India aspires to build a future‐ready healthcare system that is inclusive, technology‐driven and resilient [21]. Global models such as Singapore’s smart ED, South Korea’s AI‐assisted dispatch and WHO’s GEM‐CARN showcase digital integration, advanced triage and governance frameworks that India can adapt to the local context [22–24].

Extending electronic health records (EHR) can create new protocols for prehospital care staff, enhancing efficiency, improving patient outcomes and facilitating personalised care. This will benefit prehospital care staff and other stakeholders, such as police, fire teams, insurance agencies and government officials [25], enabling urgent retrieval of medical records during emergencies. The qualitative insights from this study inform the development of awareness programs and technological solutions for prehospital emergency care in cities.

Faster and safer patient transport supported by digital public awareness initiatives and citizen training is essential; however, progress depends on overcoming financial, behavioural and mindset barriers. Training and public awareness programs can be implemented through media campaigns, community workshops and educational initiatives [25, 26], while a more comprehensive understanding of human factors is crucial for successful technology implementation [27]. Together, these measures can strengthen prehospital care by ensuring uninterrupted coverage, enabling tracking and identification of unknown patients and improving treatment at the scene and en route, supported by continuous updates and a well‐designed ambulance that builds value and public trust.

Clear medical protocols for healthcare staff and guidelines for citizens using digital tools can improve consistency, while hybrid technology‐based simulations strengthen paramedic skills for complex emergencies. Training should be responsive to future challenges [28, 29], with opportunities for enhanced professional development incorporating innovative tools, ethical data use and emerging technologies such as advanced AR/VR, AI, digital literacy and interdisciplinary modules. Building on these staff‐perceived opportunities and insights from the broader EMS literature, the authors propose that national and state guidelines supporting telemedicine, AI‐assisted triage and seamless hospital–ambulance data exchange are central to prehospital care transformation, subject to relevant stakeholder validation.

While the findings broadly align with the EMS Agenda 2050 vision [14], participants also highlighted several gaps between its guiding principles (#_) and current EMS realities in India. Consistent with the principles of inherently safe and effective care (#1) and socially equitable care (#4), participants described limited public awareness and financial constraints as barriers to timely and appropriate emergency response. Challenges associated with integrated and seamless (#2) and reliable and prepared (#3) care were reflected in bystander interference, delays in decision‐making and uncertainty during emergencies. Participants further highlighted inconsistencies across the prehospital pathway, indicating gaps in sustainable, efficient care (#5), while emphasising the need for context‐sensitive digitalisation and innovation to support adaptable, innovative care (#6). Together, these findings suggest that achieving the EMS Agenda 2050 vision in India will require addressing both system‐level constraints and community‐level factors through locally adapted implementation strategies.

Addressing these gaps will require locally adapted strategies that leverage emerging technologies, national digital health initiatives such as the Ayushman Bharat Digital Mission (ABDM) and 5G‐enabled electric ambulances under the PM E‐Drive scheme to improve the accessibility, coordination and equity of prehospital care in India.

### 4.3. Interdependence of Human Factors and Technology Adoption

Technologies cannot be evaluated in isolation from lived work realities. Participants’ recurring emphasis on diverse factors suggests a need for realignments, as everyday practices shape the design of digital products and technologies and their perceived usefulness. The importance of context‐responsive design and reliance on human abilities to make situational decisions is crucial. Training modules should build such competencies as future‐ready skills for prehospital care staff, supported by a robust policy framework recognising human variability.

Prehospital care staffs’ground‐level experiences shape how technologies can be adapted in smart‐city EMS. This is an initial study on this topic. The themes reported in this study highlight context‐specific barriers and opportunities that require deeper investigation in future research. This current study was focused on the experiences of prehospital staff, including technology use. Future research should consider barriers to the adoption of new technologies, including their use in workflows, and precise technical, infrastructure and human factor barriers in the context of LMICs.

Codesign with professionals can spark creative technological solutions beyond efficiency, while multistakeholder collaboration is essential for real‐world innovation [27]. Further research should explore these insights through speculative design to envision future healthcare systems.

### 4.4. Limitations

This research has some limitations. The first author’s background in industrial design and healthcare futures research may have influenced the interpretation of findings despite the use of reflexive practices throughout the study. As all interviews were conducted online during the COVID‐19 pandemic, participants joined from varying locations and contexts that may have influenced engagement and participation. The virtual format may also have limited access to nonverbal cues and interpersonal dynamics, potentially affecting the richness of data and interpretation of participants’ experiences during TA. Online interviews can limit rapport‐building, extended conversational engagement, spontaneous reflection on daily practices and reduced capability to capture nonverbal and other cues, potentially affecting the depth of responses. A subset of prehospital professionals were unable to participate in the study due to personal circumstances and busy work schedules. Although participants did not explicitly discuss gender‐related differences in care delivery, the predominantly male sample reflected the gender composition of the Indian EMS workforce. As gender may influence professional experiences, workplace interactions and emergency response practices, these perspectives warrant further exploration in future research.

The current study only reports on findings from interviews with these prehospital professionals. Participants were recruited from across private, public and small‐scale ambulance service operators. It is acknowledged that there is the potential for selection bias in this study. Future studies could address this through stratified sampling. Separate research by the authors is addressing the perspectives of citizens and other stakeholders.

The study prioritised sampled prehospital professionals to foreground end‐user realities influencing technology use at this stage. Other important stakeholders from the digital ecosystem, such as IT and health tech developers, should be considered in a separate study. Further research should also investigate and involve nonmedical emergency professionals, including police officers, fire attendants and disaster management rescue teams, who play crucial roles in health emergencies.

## 5. Conclusions and Recommendations

This qualitative study demonstrated that prehospital care staff manage complex emergency scenes with empathy and professionalism while deriving a strong sense of purpose. Participants expressed confidence in their hands‐on expertise and described the adaptive use of smartphones for effective communication and data exchange. Collectively, the findings indicate that future prehospital care systems require workflow redesign to create an efficient service, aligned with technology‐enabled training and practice.

Based on these findings, the authors argue that strengthening prehospital care requires stakeholder collaborations, active engagement from policymakers and healthcare leaders, citizen awareness initiatives that foster proactive communities, and context‐specific strategies for integrating emerging technologies into smart‐city EMS.

The findings emphasise the importance of developing newer protocols, digital training resources and technology‐enabled service models to enhance prehospital care; however, the feasibility, effectiveness and implementation of such approaches need further investigation.

This study contributes to a growing body of research examining the future prehospital care from multiple stakeholder perspectives. Ongoing work by the authors extends these findings by exploring speculative EMS futures and incorporating citizens’ and other stakeholders’ perspectives to inform contextually relevant innovations in emergency care.

## Funding

This research did not receive any specific grant funding.

## Disclosure

The authors acknowledge the use of Microsoft 365 Copilot 3.3.19 (accessed July/2026) only to improve readability and language. All AI‐generated content was reviewed and revised by the authors, who take full responsibility for the accuracy and integrity of the work.

## Conflicts of Interest

The authors declare no conflicts of interest.

## Data Availability

The research data generated and analysed during this study are not publicly available as it includes sensitive or confidential information but are available from the corresponding author on reasonable request.
